# Cognitive Bias Modification Using Mental Imagery for Depression: Developing a Novel Computerized Intervention to Change Negative Thinking Styles

**DOI:** 10.1002/per.855

**Published:** 2011-11-18

**Authors:** TAMARA J LANG, SIMON E BLACKWELL, CATHERINE J HARMER, PHIL DAVISON, EMILY A HOLMES

**Affiliations:** Department of Psychiatry, University of OxfordUK

**Keywords:** cognitive bias modification, mental imagery, depression, computerized interventions, treatment innovation

## Abstract

Why do some people see their glass as half-empty rather than half-full or even imagine that the glass will be filled in the future? Experimental methods can illuminate how individual differences in information processing style can profoundly impact mood or even result in disorders such as depression. A computerized cognitive bias modification intervention targeting interpretation bias in depression via positive mental imagery (CBM-I) was evaluated by investigating its impact on mental health and cognitive bias compared with a control condition. Twenty-six depressed individuals completed either positive imagery-focussed CBM-I or a control condition daily at home over one week. Outcome measures were collected pre-treatment and post-treatment and at two-week follow-up. Individuals in the positive condition demonstrated significant improvements from pre-treatment to post-treatment in depressive symptoms, cognitive bias and intrusive symptoms compared with the control condition. Improvements in depressive symptoms at two-week follow-up were at trend level. The results of this first controlled comparison of positive imagery-focussed CBM-I for depression further support the clinical potential of CBM-I and the development of a novel computerized treatment that could help patients imagine a more positive future. Broader implications concern the modification of individual differences in personality variables via their interaction with key information processing targets. Copyright © 2011 John Wiley & Sons, Ltd.

Cognitive theories of depression emphasise the role of negative cognitive biases in the development and maintenance of the disorder (Beck, 1976). These include a negative interpretation bias – the tendency to interpret ambiguous information in a negative way (Butler & Mathews, 1983; Rude, Wenzlaff, Gibbs, Vane, & Whitney, 2002). Depression is also characterised by biases in memory (Mathews & MacLeod, 2005) and attention (e.g. Joormann & Gotlib, 2007) as well as reduced vividness for positive imagery of both the future (Morina, Deeprose, Pusowski, Schmid, & Holmes, 2011) and the past (Werner-Seidler & Moulds, 2011). There has been increasing interest in trying to understand the contributions of these various biases in information processing to the clinical disorder (Gotlib & Joormann, 2010; Koster, De Lissnyder, Derakshan, & De Raedt, 2011). Although such biases are common across many emotional disorders, the nature of these biases varies depending upon the disorder (for a review, see Mathews & MacLeod, 2005). For example, in anxiety, an attentional bias towards threat-related material is well established, whereas in depression, an attentional bias towards negative self-referent (mood-congruent) material has been demonstrated (Baert, De Raedt, & Koster, 2010).

Psychological theories of depression take a continuum approach in which the negative processing styles that characterise the disorder are conceptualised as existing at the extreme end of a continuum of processing styles that show considerable variation throughout the general population. Taking a continuum approach, the personality trait of neuroticism, for example, may be seen as a vulnerability factor for depression. By linking this approach with a mechanism approach, we aim to better understand the underlying information processing biases that might contribute to the clinical disorder and personality traits.

The personality trait neuroticism refers to ‘temperamental sensitivity to negative stimuli’ (p. 247; Enns & Cox, 1997). It is associated with emotional instability, low self-esteem and negative emotional experiences (Beratis, Rabavilas, Papadimitriou, & Papageorgiou, 2011). It is highly predictive of subsequent major depression and has been suggested to index genetic risk for this illness (Kendler, Gatz, Gardner, & Pederse, 2006). Consistent with this, volunteers who score high on this dimension show increased processing of negative compared with positive emotional information in cognitive and neurocognitive measures even in the absence of current or prior depressive illness (Chan, Goodwin, & Harmer, 2007; Chan, Harmer, Goodwin, & Norbury, 2008; Chan, Norbury, Goodwin, & Harmer, 2009). These results suggest that negative biases in emotional processing may be part of the complex cognitive style of individuals at high risk of developing depression. Of particular interest are observations that these negative biases in high neuroticism volunteers can be remediated by antidepressant drug administration even before changes in mood or subjective state are seen (Di Simplicio, Norbury, & Harmer, 2011).

Although a personality trait such as neuroticism illustrates the continuum between the healthy population and those who suffer from emotional disorders such as depression, it can be helpful to break down such broad traits into specific components such as individual cognitive biases in order to elucidate mechanisms implicated in the development and maintenance of depression. Such biases also exist on a continuum in the general population and can be measured by using experimental psychology laboratory tasks. For example, in relation to interpretation bias, Rude et al. (2002) administered a measure of interpretation bias (the Scrambled Sentences Test) to undergraduate students and found that scores on it not only correlated with self-report symptoms of depression but that scores on the task when completed under the condition of ‘cognitive load’ (remembering a six-digit number), in fact, predicted symptoms of depression four to six weeks later. In relation to a deficit in generating positive imagery about the future, Holmes, Lang, Moulds, and Steele (2008) found that low mood in healthy participants was associated with low ratings of vividness of positive future imagery on the Prospective Imagery Task (PIT; Stöber, 2000). Similarly, in a clinical study, participants with major depressive disorder generated significantly less vivid imagery on the PIT than a healthy control group (Morina et al., 2011).

Our understanding of the causal role of information processing biases in the development of individual differences in depression and trait anxiety has been greatly enhanced in recent years by the increased use of a set of experimental paradigms referred to as ‘cognitive bias modification’ (CBM; Koster, Fox, & MacLeod, 2009). These are procedures designed to directly target information processing biases in order to observe the effects on subsequent mood and behaviour, and they therefore allow a test of causal hypotheses concerning the contributions of selective information processing to individual differences. CBM methodology was initially developed to assess whether interpretation and attentional biases were causal in the development of individual differences such as trait anxiety. Mathews and Mackintosh (2000), for example, tested whether inducing a negative interpretation bias using a CBM technique could lead to changes in anxious mood. Further, such techniques were used to assess the impact of these biases on vulnerability to these disorders, assessing the impact of bias modification on emotional reactivity (Hoppitt, Mathews, Yiend, & Mackintosh, 2010; MacLeod, Koster, & Fox, 2009). In the context of individual differences such as depressive tendencies, CBM was further developed not only to test hypotheses derived from traditional cognitive theories of depression but also to examine the contribution of a range of other cognitive biases (e.g. attentional bias) (Baert, De Raedt, Schacht, & Koster, 2010). Recent studies have even started to use CBM to investigate the relationship between different biases such as interpretation and memory (Salemink, Hertel, & Mackintosh, 2010; Tran, Hertel, & Joormann, 2011) and to study cognitive biases in children or adolescents (e.g. Lothmann, Holmes, Chan, & Lau, 2011; Vassilopoulos, Blackwell, Moberly, & Karahaliou, 2012).

The success of CBM in modifying biases has highlighted its potential not only in testing and developing the psychological theory underpinning our understanding of emotional disorders but also in developing new innovative treatments. The biases in information processing that characterise depression are a central target of current psychological treatments such as Cognitive Behavioural Therapy (CBT) for depression, a leading evidence-based treatment option (e.g. Hollon, Thase, & Markowitz, 2002). However, people suffering from depression still struggle to access effective psychological treatments, and the disorder is increasingly prevalent and costly, with the World Health Organization ranking it as the leading cause of burden of all diseases in middle and high-income countries (World Health Organisation, 2004). This has led to increasing calls for the development of innovative treatments deliverable via computer or telephone (Marks & Cavanagh, 2009; Simon & Ludman, 2009). Recent innovations have included the adaptation of CBT for computerized delivery via the internet (e.g. Andersson et al., 2005). As most CBM approaches are delivered via computer, they lend themselves particularly well to development into novel accessible interventions. CBM approaches to depression to date have targeted memory (Joormann, Hertel, LeMoult, & Gotlib, 2009; Raes, Williams, & Hermans, 2009), attention (Baert, De Raedt, Schacht, et al., 2010) and interpretation bias (Blackwell & Holmes, 2010).

In the current study, a CBM methodology was deployed to the theoretical prediction that selective processing styles (in this case, when faced with ambiguity, the inability to generate positive interpretations) underpin the disposition to experience clinical symptoms of depression. The paradigm targeted this interpretation bias via positive mental imagery and originated in the work by Mathews and Mackintosh (2000). In this paradigm, individuals are repeatedly presented with ambiguous scenarios whose interpretation is constrained in either a positive or negative way, with the aim of training the corresponding bias. Crucially, participants have to imagine themselves in the scenarios presented. Subsequent studies employing this paradigm have highlighted the importance of generating positive mental imagery in the effectiveness of this CBM (Holmes, Coughtrey, & Connor, 2008; Holmes, Lang, & Shah, 2009; Holmes, Mathews, Dalgleish, & Mackintosh, 2006). The use of mental imagery in modifying interpretation bias was originally developed to test the hypothesis that imagery has more powerful effects on emotion than verbal processing (Holmes & Mathews, 2005). However, with the development of CBM for depression, the use of imagery in this paradigm is of particular importance. Depressed mood and major depressive disorder are associated with a deficit in generating positive imagery about the future (Holmes, Lang, et al., 2008; Morina et al., 2011). As imagining the outcome of a situation may be one particularly powerful way of resolving ambiguity, an inability to generate positive imagery may make a particularly toxic contribution to interpretation bias, as it may limit the possibility of generating positive interpretations (Holmes, Lang, & Deeprose, 2009). Furthermore, an inability to imagine a positive future is likely to contribute significantly to the hopelessness that characterises depression (Beck, Weissman, Lester, & Trexler, 1974). Therefore, the requirement to repeatedly practise generating positive imagery in response to ambiguous stimuli in this CBM paradigm may make it particularly suitable for clinical application in depression. We believe that generating future imagery is inherent in this paradigm, as the resolution of the ambiguous start requires prospective cognition to complete the outcome of the scenarios.

Blackwell and Holmes (2010) carried out an initial investigation of computerized CBM targeting interpretation via positive mental imagery in depression. In this study, conducted as a single case series, seven participants currently experiencing a major depressive episode completed first a ‘baseline’ week (daily measures of mood and cognitive bias), then an ‘intervention’ week in which they completed a session of CBM each day. Overall, the group showed large effect sizes for improvements in depressive symptoms, cognitive bias and general mental health. Improvements in depressive symptoms were maintained at two weeks. The case series allowed participant feedback to be used to tailor the laboratory intervention for clinical use.

Although these results provided some initial support for the potential of this positive imagery-focussed CBM-I for depression, a pilot study with a control comparison condition is needed as an important next step. The current study therefore aimed to compare the impact of this CBM-I in major depressive disorder with a control condition over the same time period (one-week intervention plus two-week follow-up) as a precursor to investigating the impact of the programme over a longer time period in future studies. For a robust test, the control condition was designed to be as close to the experimental condition as possible; that is, instead of exclusively positive CBM stimuli, stimuli of mixed valence (half positive and half negative) were used (cf. Amir, Beard, Burns, & Bomyea, 2009). As the importance of generating imagery for the success of the CBM paradigm had been well established in previous studies with healthy volunteers (Holmes et al., 2008; Holmes, Lang, & Shah, 2009; Holmes et al., 2006), we did not aim to test this again in this clinical translational study, and thus, identical imagery instructions were used for both conditions. In other words, the focal variable was the induction of a positive interpretational tendency via mental imagery, and as such, the control condition was designed with this in mind.

The imagery-focussed CBM-I was deployed along the following lines. After a first session in the laboratory, participants completed daily sessions for one week with the programme installed on a computer in their home. Following feedback about the repetitive nature of the task in Blackwell and Holmes (2010), three different types of stimuli were used to provide variation and maintain interest. These were the CBM as used by Blackwell and Holmes (2010), with imagery generation of interpretations via auditory stimuli (‘IGen-Auditory’), an imagery generation of interpretations via pictorial stimuli (‘IGen-Picture’) CBM as described by Holmes et al. (2008) and a CBM of appraisals (Lang, Moulds, & Holmes, 2009). Each daily session of the intervention comprised 64 stimuli in total, arranged into eight blocks of eight after Salemink et al. (2009).

An interesting suggestion from the study by Blackwell and Holmes (2010) was that there may be differences in ability to engage in imagery between those who benefit from completing the CBM task (who they classified as ‘responders’) and those who do not benefit (who they classified as ‘non-responders’). However, this suggestion arose from qualitative feedback from participants as imagery ability was not measured. It would be helpful from both a theoretical and a treatment development perspective to be able to identify characteristics of those people who may or may not gain a benefit from completing a CBM programme, particularly as the ‘responders’ and ‘non-responders’ appeared to have qualitatively different experiences of the CBM. Thus, by administering measures of imagery ability pre-treatment, the study aimed to enable comparison of ‘responders’ and ‘non-responders’ within the positive condition on baseline imagery ability.

The current pilot study therefore aimed to extend previous findings by investigating the impact of repeated sessions of imagery-focussed CBM-I in a sample of participants experiencing a current major depressive episode when compared with a control group receiving a control version of the same programme. The study was a first application of this ‘multi-component’ CBM combining IGen-Auditory, IGen-Picture and CBM of appraisals. Although the main outcomes measured were symptoms of depression and negative cognitive biases, the study additionally aimed to investigate the impact of the CBM-I on trait anxiety. Depression and anxiety are highly comorbid (Brown, Campbell, Lehman, Grisham, & Mancill, 2001), and anxiety is also characterised by a negative interpretation bias (Mathews & MacLeod, 2005) although evidence for a link between lack of positive imagery and anxiety is mixed (see Morina et al., 2011). We may therefore expect the CBM-I programme to have a significant impact on trait anxiety as well as depression. Finally, the current study can help provide an understanding of the causal role of interpretation biases in individual differences in depression and anxiety specifically with respect to atypically severe symptoms of depression.

Our hypotheses were as follows:

## Symptoms

*Hypothesis 1a:* Participants assigned to positive imagery-focussed CBM-I (‘positive condition’) would demonstrate greater reductions in measures of depressive symptoms from pre-treatment to post-treatment than a control group that completed a control version of the same programme (‘control condition’).

*Hypothesis 1b:* Participants in the positive condition would demonstrate a greater reduction in general levels of anxiety than those in the control condition from pre-treatment to post-treatment.

*Hypothesis 1c:* Participants in the positive condition would demonstrate a greater reduction in negative intrusive memories than those in the control condition from pre-treatment to post-treatment.

*Hypothesis 1d:* At two-week follow-up, participants in the positive condition would demonstrate greater reductions in symptoms of depression and general levels of anxiety than participants in the control condition.

## Negative cognitive bias

*Hypothesis 2:* Participants in the positive condition would demonstrate a greater reduction in negative cognitive bias than those in the control condition from pre-treatment to post-treatment.

## Clinical impact and possible mechanisms

*Hypothesis 3a:* A greater proportion of participants in the positive condition would show clinically significant change in symptoms of depression at post-treatment and follow-up than in the control condition.

*Hypothesis 3b:* Within the positive condition, the reduction in symptoms of depression over the one-week intervention would correlate with the reduction in negative cognitive bias over this time.

*Hypothesis 3c:* Within the positive condition, compared with ‘non-responders’, those classed as ‘responders’ to the intervention at follow-up would score higher on baseline imagery.

## METHOD

### Participants

Participants were recruited through clinicians in the local health service and poster advertisements in the local area. Participants were eligible if they met the criteria for a current major depressive episode based on the Diagnostic and Statistical Manual of Mental Disorder 4th Edition (DSM-IV) criteria using the structured clinical interview for DSM-IV Axis I disorders (SCID-I; First, Spitzer, Gibbon, & Williams, 1996), administered by the first author, in the absence of current substance abuse, suicidality, psychological treatment, psychosis, recent change in medication or history of bipolar disorder. Forty individuals attended an assessment session. Of these, 10 did not meet inclusion criteria, and two were too busy to participate further. Twenty-eight individuals in total were therefore allocated to an experimental condition. Participants were randomised to the positive or control condition by using a computerized random number generator following their assessment.

Two participants dropped out after the first session of CBM-I. Twenty-six participants therefore completed post-treatment measures, and 25 completed follow-up measures at two-week post-treatment. Demographic information for the participants is presented in Table 1.

**Table:** Sample characteristics

	Positive condition	Control condition
(*n* = 13)	(*n* = 13)
Age (years), *M* (*SD*)	30.2 (11.5)	26.7 (6.2)
Gender (%)		
Female	70	85
Male	30	15
Years of education, *n*		
11 or fewer	1	1
12–13	1	2
14–15	3	4
16–17	6	1
More than 17	2	5
Previous psychological treatment, *n*	2	2
Currently taking antidepressants, *n*	5	3
SUIS, *M* (SD)	3.33 (0.65)	3.45 (0.58)
PIT		
Positive	24.23 (10.18)	30.62 (8.12)
Negative	29.77 (10.59)	34.62 (7.43)

*Note*. SUIS = Spontaneous Use of Imagery Scale; PIT Positive/Negative = Prospective Imagery Task, Positive/ Negative items.

### Intervention

#### Overview

There were seven sessions of imagery-focussed CBM-I completed daily over the course of one week. The multi-component CBM-I included IGen-Auditory on days 2, 5 and 7 (Blackwell & Holmes, 2010), IGen-Picture on days 3 and 6 (Holmes, Mathews et al., 2008) and CBM of appraisals on day 4 (Lang et al., 2009). The first session, completed at the research centre in the presence of the researcher, comprised sets of stimuli from all three CBM-I components. Participants completed all subsequent sessions on a computer at home. The CBM-I was presented using E-Prime software (Version 2.0, Pittsburgh: Psychology Software Tools Inc.). The schedule of the imagery-focussed CBM-I was designed such that participants never completed the same kind of task on two consecutive days in order to increase participant engagement. As only the IGen-Auditory task had been tested previously with a depressed sample, the schedule included more sessions of this task than the other two tasks.

#### IGen-Auditory

Each session of IGen-Auditory comprised 64 training scenarios, grouped into eight blocks of eight paragraphs as in Blackwell and Holmes (2010). An additional 24 paragraphs were presented in the first session. There were thus 216 different positive training paragraphs in total. Paragraphs lasted 10 to 13 s and were digitally recorded. They were presented stereophonically via headphones. Presentation of each paragraph was followed by a 2 s pause and a beep to prompt participants to open their eyes.

The paragraphs were designed such that they started ambiguous as to their potential outcome, which only became clear towards the end of the statement. For example: ‘You ask a friend to look over some work you have done. They come back with some comments, which are *all very positive*’ (positive resolution in italics) versus ‘You ask a friend to look over some work you have done. They come back with some comments, which are *all highly critical*’ (negative resolution in italics). In the positive condition, every training paragraph resolved positively, whereas in the control condition, half resolved positively and half resolved negatively. Thus, in the positive condition, a specific learning contingency was established between the ambiguous start of the scenario and a positive resolution, whereas in the control condition, no such contingency was established. Participants were instructed to ‘imagine the scenarios as if you are actively involved, seeing them through your own eyes’. To focus participants on generating imagery (Holmes et al., 2006), after each training paragraph, they rated the vividness of their imagery (‘How vividly could you imagine the situation that was described?’) on a 5-point (1–5) scale ranging from ‘*not at* all’ to ‘*very*’.

#### IGen-Picture

Each session of IGen-Picture comprised 64 picture-word combinations grouped into eight blocks of eight. An additional 24 stimuli were presented at the first CBM-I session. There were thus 152 picture-word stimuli in total, developed from previous studies (Holmes, Mathews et al., 2008). These were colour photographs of neutral everyday stimuli with dimensions of approximately 640 × 480 pixels, displayed on the computer screen. Each picture was combined with a word or short phrase, which provided a potential positive or negative interpretation of the picture (e.g. a picture of a street full of shoppers accompanied by the word ‘lively’ for a positive valence or by the word ‘intimidating’ for a negative valence). Participants were instructed to combine the picture and word cues to form a mental image. The same picture stimuli were used in both the positive and the control condition with only the word combination changing. In the positive condition, participants were repeatedly presented with positive words, whereas in the control condition, half of the combinations were positive and half were negative.

Each picture-word combination was presented for 3 s followed by a black screen displaying ‘Close your eyes and imagine’ for 3 s. A beep then signalled for participants to open their eyes and rate how vividly they could imagine the combination of the picture and word.

#### CBM of appraisals

The CBM of appraisals session included 64 stimuli, presented in eight blocks of eight. An additional 16 were presented at the first treatment session. The CBM of appraisals was derived from Lang et al. (2009). The stimuli were derived from a range of maladaptive cognitions listed on the Response to Intrusions Questionnaire (Clohessy & Ehlers, 1999). In the positive condition, all sentences resolved positively (e.g. ‘having an intrusive memory means nothing is wrong with me’), whereas in the control condition, half resolved positively and half resolved negatively (e.g. ‘having an intrusive memory means something is wrong with me’).

Participants were to imagine themselves in the situations described by the statements. Statements always appeared on the screen in two parts. The first half appeared on the screen for 2 s, followed by a presentation of the remainder of the statement (in the form of a word fragment, e.g. ‘n_th_ng is w_ong w_th me’). Participants were asked to press the advance key when they knew what the first missing letter was and to type it in. The correct word then appeared on the screen.

The CBM of appraisals was included, as negative intrusive memories are common across a range of mental health problems (Holmes, Arntz, & Smucker, 2007; Holmes & Hackmann, 2004; Holmes & Mathews, 2010), and up to 90% of depressed individuals have been found to report negative intrusive memories (Birrer, Michael, & Munsch, 2007). Such memories have been suggested to play an important role in maintaining depressed mood (Patel et al., 2007), and in fact, initial work on targeting negative intrusive memories in depression in the context of cognitive behavioural therapy has found promising results (Wheatley et al., 2007). The CBM of appraisals developed by Lang et al. (2009) was designed to target the negative appraisals of intrusive memories (e.g. *having intrusive memories means I'm crazy*) that may play a key role in the distress they cause.

### Baseline measurement

In addition to basic demographic information, the following baseline measures of imagery use were administered pre-treatment:

#### Spontaneous Use of Imagery Scale (SUIS; Reisberg, Pearson, & Kosslyn, 2003)

The SUIS was used as a measure of participants' current everyday use of imagery. The questionnaire consists of 12 items, for example: ‘When I think about a series of errands I must do, I visualise the stores I will visit’. Each item is rated on a 5-point scale (1 = *never appropriate* and 5 = *always completely appropriate*). Reisberg et al. (2003) report excellent internal consistency, α = 0.98.

#### Prospective Imagery Task (PIT; Holmes, Lang et al., 2008; Stöber, 2000)

The PIT is a measure of ability to generate mental imagery about future events. As in Holmes et al. (2008), participants were asked to form a mental image of 10 negative future scenarios and 10 positive future scenarios. These included events such as ‘You will have a serious disagreement with your friend’ or ‘You will do well on your course’. Each image was rated for vividness on a continuous 5-point Likert scale (1 = *no image at all* and 5 = *very vivid*). As internal consistency for the PIT was not reported previously, we calculated it for this sample. For the positive items, α = 0.92 (excellent) and for the negative items, α = 0.87 (good).

### Outcome Measurement

The following outcome measures were completed at the research centre before and after the one-week CBM intervention:

#### Measures of symptoms

##### Beck Depression Inventory - second edition (BDI-II; Beck, Steer, & Brown, 1996)

The BDI-II is a widely used self-report measure of depressive symptoms with robust reliability and validity (Beck et al., 1996). Scores are classified as follows: 0–13 = minimal depression; 14–19 = mild depression; 20–28 = moderate depression; 29–63 = severe depression (Beck et al., 1996). An excellent internal consistency is reported for an outpatient sample, α = 0.92 (Beck et al., 1996).

##### Hamilton Rating Scale for Depression (HRSD; Hamilton, 1960)

The 17-item version of the HRSD interview is an interview-based assessment tool that is commonly used in pharmacological studies of depression to measure severity of depressive symptoms. It was therefore included in addition to the BDI-II in order to allow comparison with both psychological and pharmacological literature, as in a number of recent trials of psychological therapies for depression (Cuijpers, van Straten, Bohlmeijer, Hollon, & Andersson, 2010). The HRSD possesses high reliability and validity, and good internal consistency is reported, α = 0.82 (Potts, Daniels, Burnam, & Wells, 1990; Williams, 1988). Interviews were conducted by the first author at pre-treatment and post-treatment. A subset of participant interviews (*n* = 6) were randomly selected from each condition (*n* = 3). These were rated by an independent rater who was blind to participant condition. Average agreement between the experimenter and the independent rater was 94.4% for pre-treatment scores and 93.2% for post-treatment scores, indicating good inter-rater reliability (Barker, Pistrang, & Elliot, 1996).

##### Spielberger State- Trait Anxiety Inventory – Trait version (STAI-T; Spielberger, Gorsuch, Lushene, Vagg, & Jacobs, 1983)

The trait version of the STAI was used as an index of general levels of anxiety, in line with other recent studies investigating the effects of repeated sessions of CBM paradigms (Brosan, Hoppitt, Shelfer, Sillence, & Mackintosh, 2011; Mathews, Ridgeway, Cook, & Yiend, 2007; Salemink et al., 2009). Participants were asked to rate 20 anxiety related statements for how relevant they are to how they ‘generally feel’ on a 4-point scale (‘*almost never*’, ‘*sometimes*’, ‘*often*’, ‘*always*’). This scale is reported to have satisfactory reliability and validity with excellent internal consistency reported, α > 0.90 (Spielberger et al., 1983).

##### Impact of Event Scale (IES; Horowitz, Wilner, & Alvarez, 1979)

The eight-item intrusions subscale of the IES was used to measure intrusive symptoms, as such negative intrusive memories are common in depression and thought to play an important role in maintaining depressed mood (Birrer et al., 2007; Patel et al., 2007). Although more commonly used in the context of post-traumatic stress disorders, the IES has also been previously used an index of intrusive negative memories in depression (Lang et al., 2009). Example items include ‘Pictures about it popped into my mind’. Items are rated on a 4-point scale (*not at all* = 0, *rarely* = 1, *sometimes* = 3, *often* = 5). A recent meta-analysis reports good internal consistency for the intrusions subscale, α = 0.86 (Sundin & Horowitz, 2002).

#### Measures of negative cognitive bias

##### The Scrambled Sentences Test (SST; Rude et al., 2002)

The SST was used as a measure of depressive interpretation bias, as targeted by the IGen-Auditory and IGen-Picture paradigms. Participants unscrambled a list of 20 scrambled sentences (e.g. winner born I am loser a) under a cognitive load (remembering a six-digit number). This measured the tendency of participants to interpret ambiguous information either positively (I am a born winner) or negatively (I am a born loser). A ‘negativity’ score is generated by calculating the proportion of sentences completed correctly with a negative emotional valence. Rude et al. (2002) found scores on the SST to predict depressive symptoms four to six weeks later. Two sets of 20 scrambled sentences were used in the current experiment, one administered pre-treatment and the other post-treatment.

##### Response to Intrusions Questionnaire (RIQ; Clohessy & Ehlers, 1999)

The negative appraisal subscale of the RIQ was used to assess appraisal bias for intrusive memories, that is, the cognitive bias targeted by the CBM of Appraisals paradigm. As in Starr and Moulds (2006), participants were asked about a negative intrusive memory they had experienced in the past week. Participants then responded to six items assessing negative appraisals of intrusions. Ratings were made on a 7-point (1–7) scale ranging from ‘*totally disagree*’ to ‘*totally agree*’. This subscale is reported to have good internal consistency as reported in Starr and Moulds (2006), α = 0.84.

### Follow-up

Following Blackwell & Holmes (2010), two weeks after completing the post-treatment measures, participants again completed the BDI-II, and in addition, the STAI-T, online or by post. The self-report nature of these questionnaire measures means that they are suitable for online follow-up, in contrast to interviewer-rated measures such as the HRSD, which require face-to-face assessment.

### Statistical analysis

Baseline characteristics of participants in the positive and the control conditions were compared using two-tailed independent samples *t* tests or chi-square tests of independence. Mixed ANOVAs with within-subjects factor of time (pre-treatment vs. post-treatment or pre-treatment vs. follow-up) and between-subjects factor of condition (positive vs. control) was used to investigate the impact of the CBM-I. Significant or trend level effects were then further examined by using paired samples *t* tests. Differences in rates of clinically significant change between the positive and the control conditions were compared by using chi-square tests of independence, one-tailed in line with the study hypothesis. Participants in the positive condition were classified as ‘responders’ or ‘non-responders’ on the basis of whether they demonstrated clinically significant change on the BDI-II from pre-treatment to follow-up. This two-week time frame was chosen as the best available clinical index of therapeutic benefit from the intervention, in the absence of the single-case analysis employed by Blackwell and Holmes (2010). Differences on imagery and other baseline measures between ‘responders’ and ‘non-responders’ in the positive condition were compared by using two-tailed independent samples *t* tests or chi-square tests of independence.

## RESULTS

### Preliminary analysis

The two groups did not statistically differ with regard to demographic characteristics or any of the baseline measures, apart from the positive items of the PIT on which there was a trend for participants in the control condition to score higher than those in the positive condition, *t*(24) = 1.77, *p* = .09^1^ (see Table 1). There was no significant difference between rate of attrition following randomization to the positive and the control condition, 7.1% *v*. 7.1%; *χ^2^* (1, *n* = 28) = 0.00, *p* = 1.00. Of the participants who completed the study, all completed at least six out of a possible seven sessions, and there was no significant difference between the positive (*M* = 6.85, *SD* = 0.38) and the control (*M* = 6.77, *SD* = 0.44) conditions on the number of CBM sessions completed, *t*(24) < 1. For the vividness ratings made during IGen-Auditory sessions, there was no significant difference between the positive (*M* = 3.30, *SD* = 0.78) and the control (*M* = 3.43, *SD* = 0.49) conditions, *t*(24) < 1. Similarly, for vividness ratings made during IGen-Picture sessions, there was no significant difference between the positive (*M* = 3.40, *SD* = 0.69) and the control (*M* = 3.51, *SD* = 0.52) conditions, *t*(24) < 1. Table 2 presents the correlations between baseline measures.

**Table:** Correlations between participant characteristics and outcome measures at baseline

Measure	1	2	3	4	5	6	7	8	9	10
1. Age	_	−0.051	−0.219	−0.126	0.374	0.181	0.087	−0.223	0.288	0.267
2. SUIS		_	0.313	0.376†	−0.167	−0.029	−0.173	0.237	−0.340†	−0.278
3. PIT-P			_	0.462*	0.004	−0.095	−0.083	0.280	0.028	−0.383†
4. PIT-N				_	0.121	0.202	0.207	0.257	0.230	−0.004
5. BDI-II					_	0.634**	0.739**	0.310	0.528**	0.313
6. HRSD						_	0.487*	0.323	0.440*	0.176
7. STAI-T							_	0.343†	0.428*	0.498*
8. IES-I								_	0.134	0.049
9. SST									_	0.250
10. RIQ										_

*Note. n = 26*. SUIS = Spontaneous Use of Imagery Scale; PIT-P/ / PIT-N = Prospective Imagery Task, Positive/ Negative items. BDI-II = Beck Depression Inventory-II; HRSD = Hamilton Rating Scale for Depression; STAI-T = Trait subscale of the State–Trait Anxiety Inventory; IES-I = Intrusions subscale of the Impact of Event scale; SST = Scrambled Sentences Test; RIQ negative appraisals = Negative appraisals subscale of the Response to Intrusions Questionnaire.

†*p* < .10 *p < .05 **p < .01.

### Post-treatment outcome analysis

#### Measures of symptoms

##### Depressive symptoms

For the BDI-II, there was a significant main effect of time, *F*(1, 24) = 7.58, *p* = 0.01, η² = .24, but not of condition, *F*(1, 24) = 1.07, *p* = 0.31. Consistent with hypothesis 1a, there was a significant interaction of time with condition, *F*(1, 24) = 5.53, *p* = 0.03, η² = .19. Similarly for the HRSD, there was a significant main effect of time, *F*(1, 24) = 8.00, *p* = 0.009, η² = .25, but not of condition, *F*(1, 24) = 2.51, *p* = 0.13, and there was a significant interaction of time with condition, *F*(1, 24) = 13.23, *p* = 0.001, η² = .36. Within the positive condition, there was a significant decrease from pre-treatment to post-treatment in both the BDI-II, *M* = 6.85, *SD* = 7.22, *t*(12) = 3.42, *p* = .005, *d* = 0.89, and the HRSD, *M* = 4.92, *SD* = 4.21, *t*(12) = 4.21, *p* = .001, *d* = 1.24. By contrast, within the control condition, there was no significant decrease in the BDI-II, *M* = 0.54, *SD* = 6.44, *t*(12) < 1, or HRSD, *M* = −0.62, *SD* = 3.52, *t*(12) < 1; see Table 3.

**Table:** Outcome measures at pre-treatment, post-treatment, and follow-up

	Pre-treatment	Post-treatment	Follow-up
	*M*	*SD*	*M*	*SD*	*M*	*SD*
BDI-II						
Positive condition	25.85	7.66	19.00	10.73	18.62	12.86
Control condition	26.46	11.15	25.92	9.66	23.08	9.77
HRSD						
Positive condition	14.92	3.97	10.00	5.85	–	–
Control condition	15.62	7.29	16.23	6.01	–	–
STAI-T						
Positive condition	62.69	6.06	55.00	9.31	54.31	9.66
Control condition	61.31	10.61	57.69	9.31	55.92	10.74
IES intrusions						
Positive condition	21.00	7.00	12.38	5.55	–	–
Control condition	21.08	6.91	18.54	5.87	–	–
SST						
Positive condition	0.47	0.22	0.33	0.22	–	–
Control condition	0.46	0.22	0.50	0.22	–	–
RIQ negative						
Positive condition	24.07	6.13	15.85	7.51	–	–
Control condition	19.92	8.60	18.00	5.37	–	–

*Note*. BDI-II = Beck Depression Inventory-II; HRSD = Hamilton Rating Scale for Depression; STAI-T = Trait subscale of the State–Trait Anxiety Inventory; SST = Scrambled Sentences Test; RIQ negative appraisals = Negative appraisals subscale of the Response to Intrusions Questionnaire; IES intrusions = Intrusions subscale of the Impact of Event scale.

##### Trait anxiety

For the STAI-T, there was a significant main effect of time, *F*(1, 24) = 24.51, *p* < 0.001, η² = .51, and no significant effect of condition, *F*(1, 24) < 1. In partial support of hypothesis 1b, the interaction of time with condition was at trend level, *F*(1, 24) = 3.19, *p* = 0.09, η² = .12. There was a significant decrease on the STAI-T within both the positive, *M* = 7.69, *SD* = 7.44, *t*(12) = 3.73, *p* = .003, *d* = 1.27, and control, *M* = 3.62, *SD* = 3.52, *t*(12) = 3.70, *p* = .003, *d* = 0.34, condition.

##### Intrusive symptoms

For the IES intrusions, there was a significant main effect of time, *F*(1, 24) = 19.88, *p* < 0.001, η² = .45, but no significant effect of condition, *F*(1, 24) = 2.08, *p* = 0.16. Consistent with hypothesis 1c, there was a significant interaction of time with condition, *F*(1, 24) = 5.90, *p* = 0.02, η² = .20. There was a significant decrease within the positive, *M* = 8.62, *SD* = 7.40, *t*(12) = 4.20, *p* = .001, *d* = 1.23, but not within the control, M = 2.5, SD = 5.16, *t*(12) = 1.78, *p* = .10, condition.

##### Measures of negative cognitive bias

For the SST, there was no main effect of time, *F*(1, 24) < 1, and no significant effect of condition, *F*(1, 24) = 1.09, *p* = 0.31, but consistent with hypothesis 2, there was a significant interaction of time with condition, *F*(1, 24) = 4.49, *p* = 0.045, η² = .16. For the RIQ, there was a significant main effect of time, *F*(1, 24) = 19.88, *p* < 0.001, η² = .45, but no significant effect of condition, *F*(1, 24) < 1. Consistent with hypothesis 2, there was a significant interaction of time with condition, *F*(1, 24) = 7.67, *p* = 0.01, η² = .24. Within the positive condition, there was a significant decrease on the SST, *M* = 0.13, *SD* = 0.16, *t*(12) = 2.92, *p* = .01, *d* = 0.60 and RIQ, *M* = 8.23, *SD* = 5.36, *t*(12) = 5.54, *p* < .001, *d* = 1.34, whereas within the control condition, neither decrease on the SST, *M* = −0.05, *SD* = 0.26, t(12) < 1 nor RIQ, *M* = 1.92, *SD* = 6.23, t(12) = 1.11, *p* = .29, was significant.

#### Follow-up

For the BDI-II, there was a significant main effect of time, *F*(1, 23) = 7.87, *p* = 0.01, η² = .26, and no significant effect of condition, *F*(1, 23) < 1. In partial support of hypothesis 1d, the interaction of time with condition was at trend level, *F*(1, 23) = 3.08, *p* = 0.09, η² = .12. The reduction was significant within the positive, *M* = 7.23, *SD* = 9.06, *t*(12) = 2.88, *p* = .01, *d* = 0.71, but not control, *M* = 1.67, *SD* = 6.46, *t*(11) < 1, condition; see Table 2. For the STAI-T, there was a significant main effect of time, *F*(1, 23) = 14.64, *p* = 0.001, η² = .39, but no significant effect of condition, *F*(1, 23) < 1. Inconsistent with hypothesis 1d, there was no significant interaction of time with condition, *F*(1, 23) = 1.74, *p* = 0.20, η² = .07.

#### Analysis of clinically significant change

On the BDI-II, clinically significant change was defined as a shift to a lower category of depressive symptom severity accompanied by a reduction greater than the reliable change index of 7.16, calculated according to the guidance provided by Jacobson and Truax (1991)^2^ and as applied by Blackwell and Holmes (2010). Consistent with hypothesis 3a, on the BDI-II, more participants demonstrated clinically significant change in the positive compared with the control condition from pre-treatment to post-treatment, 46.2% *v*. 7.7%; *p* = .04, Fisher's exact test. The equivalent difference was at trend level from pre-treatment to two-week follow-up, 53.8% *v*. 16.7%; *p* = .06, Fisher's exact test.

On the HRSD, clinically significant change is commonly defined as an improvement of 50% or greater (Hollon et al., 2002). That is, someone is said to have shown clinically significant change on the HRSD if their post-treatment score is half of their pre-treatment score or lower. Consistent with hypothesis 3a, significantly more participants demonstrated clinically significant change from pre-treatment to post-treatment on the HRSD in the positive compared with the control condition, 38.5% *v*. 0%; *p* = .02, Fisher's exact test.

#### Relationship between change in biases and symptoms of depression

To investigate whether the changes in symptoms of depression over the course of the CBM related to changes in cognitive bias (one of the hypothesised mediators), we carried out correlations between the relevant change scores. We note that the small sample size precluded formal mediation analyses (Fritz & MacKinnon, 2007). It was hypothesised that within the positive condition, changes in cognitive bias over one week would be associated with changes in symptoms of depression over that week (hypothesis 3b). Accordingly, change in score on the SST (interpretive bias) from pre-treatment to post-treatment correlated at trend level with change on the BDI-II across the same time-scale (*r* = .53, *p* = .06). Furthermore, change in score on the RIQ (interpretation bias of intrusive memories) correlated significantly with change on the BDI-II across the same timescale (*r* = .71, *p* = .006). Conversely, within the control condition, which was designed not to modify cognitive bias, neither change in score on the SST (*r* = −.24, *p* = .44) nor RIQ (*r* = .44, *p* = .13) from pre-treatment to post-treatment correlated significantly with change on the BDI-II across this timescale.

#### Comparison of ‘responders’ and ‘non-responders’ on baseline imagery

Within the positive condition, those demonstrating clinically significant change on the BDI-II from pre-treatment to follow-up (*n* = 6) were classed as ‘responders’, and those not demonstrating this clinically significant change were classed as ‘non-responders’ (*n* = 7). Consistent with hypothesis 3c, on the SUIS, responders had significantly higher scores, *M* = 3.65, *SD* = 0.64, than non-responders, *M* = 2.94, *SD* = 0.44; *t*(11) = 2.28, *p* = .04, *d* = 1.27. Consistent with hypothesis 3c, for vividness of imagery generated for the positive items of the PIT, responders scored significantly higher, *M* = 29.86, *SD* = 9.48, than non-responders, *M* = 17.67, *SD* = 6.71; *t*(11) = 2.63, *p* = .02, *d* = 1.47. For negative items of the PIT, there was no difference between vividness ratings for responders, *M* = 32.86, *SD* = 7.43 or non-responders, *M* = 26.17, *SD* = 13.20; *t*(11) < 1.

Conversely, there were no significant differences between responders and non-responders on any other pre-treatment measures or demographic characteristics (all *p* > .10), with the exception of gender. A significantly higher proportion of female participants than male participants were classed as responders, 77.8% *v*. 0%; *p* = .02, Fisher's exact test. Female participants in the positive condition generated more vivid imagery for the positive items of the PIT, *M* = 28.67, *SD* = 8.63, than male participants, *M* = 14.25, *SD* = 4.92; *t*(11) = 3.08, *p* = .01, *d* = 2.03. Female participants in the positive condition did not score significantly higher on the SUIS, *M* = 3.45, *SD* = 0.69, than male participants, *M* = 3.04, *SD* = 0.52; *t*(11) = 1.06, *p* = .31. For the vividness ratings made during the IGen-Auditory sessions, responders did not score significantly different, *M* = 3.37, *SD* = 0.85, than non-responders, *M* = 3.23, *SD* = 0.76; *t*(11) < 1. For vividness ratings made during the IGen-Picture sessions, responders did not score significantly different, *M* = 3.43, *SD* = 0.78, than non-responders, *M* = 3.36, *SD* = 0.64; *t*(11) < 1.

## DISCUSSION

This pilot study is the first to compare the impact of a CBM promoting positive interpretation via mental imagery to a closely match control condition in a sample of individuals currently experiencing a major depressive episode. Participants in the positive condition demonstrated greater improvements in depressive symptoms, intrusive images and cognitive bias than those in the control condition, supporting our hypotheses 1a,1c and 2. After just one week, approximately half the sample receiving the positive imagery-focussed CBM-I showed clinically significant change from pre-treatment to follow-up, corresponding to the proportion of ‘responders’ found in the case series by Blackwell and Holmes (2010). Interestingly, this compares to response rates found for treatments for depression, whether pharmacological or psychological (Hollon et al., 2002). These results provide further initial support for the continued development of computerized CBM as a novel intervention to target interpretation bias in depression.

In addition, the current study contributes to growing theoretical research suggesting a causal role of information processing biases in the development of individual differences in trait anxiety and depression. Specifically, the results provide some support for the contribution of an inability to generate positive interpretations to the disposition to experience atypically severe symptoms of depression. Conversely, it suggests that when faced with ambiguity, practice in forming positive images of the future may help to alleviate such symptoms. The correlational analyses suggest that within the positive condition, reduction in negative biases in interpretation and appraisal of intrusions over the one week of completing CBM were associated with reduction in depressive symptoms over this same period (hypothesis 3b). CBM paradigms were originally developed to assess the causal role of information processing biases in the development of individual differences such as trait anxiety in studies using healthy volunteers. Extending their application to clinical populations as in this study represents an important step in demonstrating that similar processes can help account for individual differences across the continuum from healthy functioning to clinical symptoms. In taking this continuum approach as outlined in the Introduction, we hope to contribute to an interweave between the experimental psychopathology and the broader literature on personality. Future avenues of research to pursue in this regard include neuroticism, but also other personality styles we know from clinical work may be associated with biases and emotional vulnerability, such as a bipolar phenotype involved in hypomanic experiences (Malik, Goodwin, & Holmes, submitted). As such, this work represents a part of what we hope becomes a tighter interdisciplinary endeavour in the future.

The current study fits within the framework of ‘experimental medicine’ (Rutter & Plomin, 2009), in that the paradigm has been developed through experimental studies informed by basic cognitive science. In contrast, the majority of current computerized interventions generally aim to reproduce more traditional face-to-face therapies or self-help by using a computer interface (e.g. Andersson et al., 2005; Christensen, Griffiths, & Jorm, 2004). CBM targets aspects of information processing hypothesised to play an important role in the maintenance of emotional disorders, namely cognitive biases. The relevance of these cognitive biases is highlighted by evidence that different treatments for depression seem to have the common effect of modifying cognitive biases (Harmer, Cowen, & Goodwin, 2011). In particular, recent evidence suggests that pharmacological treatments for depression increase the processing of positive compared with negative affective information early in treatment and before changes in mood and affect (see Harmer et al., 2011). Such effects can be seen in healthy volunteers (Harmer, Hill, Taylor, Cowen, & Goodwin, 2003; Harmer, Shelley, Cowen, & Goodwin, 2004) and in acutely depressed patients (Harmer et al., 2009), and the magnitude of these early changes in bias are associated with later therapeutic change seen with antidepressant drug treatment (Tranter et al., 2009). Such results suggest that targeting cognitive bias may be a necessary and convergent effect of different treatments for depression. Consistent with this, experimental modification of bias with training also affects cognitive bias before changes in mood are seen (Browning et al., 2011) and has a distinct neural substrate, as demonstrated in imaging studies (Browning, Holmes, Murphy, Goodwin, & Harmer, 2010). This provides convergent support for the potential promise of CBM in innovative treatment development.

However, despite the relevance of CBM for depression, much of the CBM research has tended to focus on other disorders. For example, attentional bias in emotional disorders and ways of modifying this has received much attention in recent years (Browning, Holmes, & Harmer, 2010), but the majority of this work has been carried out with relation to anxiety (e.g. Amir et al., 2009; Rinck & Becker, 2006). As the possible attentional component for each group in the current study was the same—that is, disengaging from distracting thoughts and focussing on imagery—it may be that this component contributed to the decrease in trait anxiety shown in both groups, especially given that beneficial effects of attentional training on mood have been found even with non-affective stimuli such as the sound of birds (Siegle, Ghinassi, & Thase, 2007). Similarly, although CBM targeting interpretation bias has received much attention in relation to anxiety (e.g. Hirsch, Hayes, & Mathews, 2009), the use of CBM to modify interpretation bias in depression has received surprisingly little focus despite theoretical accounts and experimental work highlighting its potential utility (e.g. Hertel & Brozovich, 2010; Holmes, Lang, & Deeprose, 2009; Joormann & D'Avanzato, 2010). The current study therefore provides an important bridging function between basic science and potential clinical application, and as such, it is particularly encouraging to obtain promising results at this stage of research.

It is notable that, consistent with hypothesis 3d, amongst those receiving the positive imagery-focussed CBM-I, everyday use of imagery and ability to generate vivid positive mental imagery at baseline differentiated significantly between those who did or did not go on to show clinically significant change in symptoms of depression from pre-treatment to follow-up (‘responders’ *v*. ‘non-responders’). This is in contrast to other clinical characteristics of the participants such as severity of depression at pre-treatment, which was no different between ‘responders’ or ‘non-responders’. However, interpretation of this is confounded by the association between gender, imagery ability and response to CBM-I within the positive group. With this small sample size, it is not possible to disentangle these associations further, and this pattern of results clearly needs replicating in a larger sample before any firm conclusions can be drawn (although cf. Steel et al., 2010, for convergent data). However, if a robust association between imagery ability and response to this imagery-focussed CBM-I could be demonstrated, this would have exciting implications in terms of being able to identify who may benefit from such a programme. It may even be that providing additional training in imagery prior to CBM could increase the proportion of people who respond. Although the current study carried out this analysis by contrasting ‘responders’ and ‘non-responders’ on imagery measures, after Blackwell and Holmes (2010), it will be useful in future studies with a larger sample to clarify whether response to such CBM programmes is best understood as a continuous or categorical variable. If, as this data suggests, generating positive imagery is useful for bringing about changes in mood, and there are individuals for whom using imagery is difficult, then there are at least two options. First, these individuals may benefit from CBM methods that do not rely on imagery (e.g. Baert, De Raedt, Schacht et al., 2010; Brosan et al., 2011; Browning et al., 2011; Joormann et al., 2009). Second, it may be possible to boost the ability of these individuals to generate imagery such that they are subsequently able to engage in imagery-based CBM (see Blackwell & Holmes, 2010). We believe the latter might be particularly important to explore given earlier findings showing that compared with verbal (non-imagery), imagery has a greater impact on positive emotion and that verbal processing of positive material can even make mood worse rather than better (Holmes, Coughtrey et al., 2008; Holmes, Lang, & Shah, 2009; Holmes et al., 2006).

The current study has a number of limitations. Most obviously, the small sample size, used here because of the pilot nature of the investigation, means that the study lacks statistical power. The small sample size also limits generalizability of the findings and precludes detailed statistical analysis of mediators or moderators of the impact of the CBM-I, and such analyses will be important in future studies in order to elucidate the mechanisms of change. This study built on Blackwell and Holmes (2010) by including a control group. In order to establish long term benefits of the CBM-I procedure, future studies will need to extend the intervention from one week (as in the current study) to several weeks, and extend the follow-up period from two weeks to several months. The trait version of the STAI, although commonly used, may not be very sensitive to changes in symptoms of anxiety over the timescale of this study. In future studies, it may therefore be helpful to include a measure that assesses anxiety symptoms over a circumscribed time such as one week, for example, the Beck Anxiety Inventory (Beck, Brown, Epstein, & Steer, 1988) or for shorter time, the state form of the STAI (Spielberger et al., 1983).

The design of the current study does not allow us to draw specific conclusions as to the exact mechanism of change associated with the CBM-I. It is possible that the effects of the treatment may be due to the promotion of positive mental imagery, changes in interpretation bias or alternatively increased positive affect resulting from exposure to the positively valence training material. Future studies that include a non-imagery control condition or a positive material condition that does not attempt to modify interpretation bias may provide a crucial comparison condition needed to determine the exact causal mechanisms. One issue for future research is to examine whether a certain baseline mental imagery may or may not be necessary for people to gain from this CBM-I paradigm. The current study suggests that engaging in positive mental imagery of future events is beneficial. However, we cannot infer from our results whether the CBM-I increased participants' underlying *ability* to generate positive imagery per se, over and above inducing positive interpretations of ambiguous situations. Thus, in future studies, it will also be helpful to measure the mechanisms of the intervention by adding additional tests of some type of underlying imagery generation ability before and after the training or even investigating transfer to alternative tasks requiring this specific ability.

Through the lens of depression, the ambiguity inherent in the small everyday occurrences that make up our lives all too easily lends itself to negative interpretations that sap motivation and fuel low mood and hopelessness. Imagine if we had something that could shape the interpretation of this ambiguity so that it instead became a rich source of positive mental imagery about the future. The imagery-focussed CBM-I in the current study aims to do just this, and moreover, is rooted in cognitive science. The low rate of drop out (7.7%) and high compliance with the CBM-I further suggests that it could be developed as a potentially viable and acceptable treatment option. In the context of increasing interest in the development of computerized interventions for depression (Marks & Cavanagh, 2009), this study provides some optimism for the potential of innovative and accessible interventions for depression and supports the ongoing investigation of imagery-focussed CBM-I as a contributor to this drive.

## Footnotes

1 In order to verify that the pattern of results found was not a consequence of this trend-level baseline difference between the two groups, we additionally ran all the analyses of outcome with PIT-positive score as a covariate. Consistent with our hypothesis that higher PIT-positive scores at baseline would be associated with a greater response to the CBM-I intervention, including this score as a covariate if anything increased the significance of the effects found in favour of the positive condition although the pattern of results was unchanged. For simplicity and for a more conservative estimate of the effects of the CBM-I, we have reported the analyses without the covariate.

2 The formula for a Reliable Change Index provided by Jacobson & Truax (1991) uses the reliability of a measure to calculate a 95% confidence level of change. The change in score from pre-test to post-test that would be unlikely to occur at *p* < .05 without actual change is given as S_diff_ × *z*, where S_diff_ is the standard error of difference between the two test scores, and *z* is the desired *z*-value (i.e. 1.96 for a significance level of *p* < .05). S_diff_ is calculated from the standard error of measurement S_E_, according to the following: S_diff_ = √[2(S_E_)^2^]. S_E_ is given by S_E_ = *s*_1_√(1 - *r*_xx_), where *s*_1_ is the standard deviation of the pre-treatment group or in small samples such as this study, the standard deviation from a standardisation sample, and *r*_xx_ is the test–retest reliability of the measure. For the current study, *s*_1_ and *r*_xx_ were taken from standardisation data (Beck et al., 1996; Steer, Brown, Beck, & Sanderson, 2001), with *s*_1_ = 9.76 and *r*_xx_ = 0.93, such that S_E_ = 2.58, S_diff_ = 3.65 and the mean change required = 7.16.
